# Gastroenterology Cases of Cutaneous Leukocytoclastic Vasculitis

**DOI:** 10.1155/2013/264189

**Published:** 2013-10-22

**Authors:** Cumali Karatoprak, Elif Arabaci, Kemal Yildiz, Mustafa Cakirca, Mehmet Ali Cikrikcioglu, Fatih Ergun, Ahmet Danalioglu, Orhan Kocaman, Hakan Senturk

**Affiliations:** Internal Medicine Clinic, Bezmialem Vakif University, Faculty of Medicine, Fatih, 34093 Istanbul, Turkey

## Abstract

Rarely, leukocytoclastic vasculitis can result from ischemic colitis, inflammatory bowel disease, and cryoglobulinemia. There is no established standard for the treatment of leukocytoclastic vasculitis associated with gastroenterologic diseases. This paper presents three cases of leukoytoclastic vasculitis, each of which is associated with a different gastroenterologic condition: ischemic colitis, Crohn's disease, and chronic hepatitis C. Each condition went into remission by treatment of leukocytoclastic vasculitis, regardless of the underlying disease.

## 1. Introduction

Vasculitis is an uncommon disease caused by destruction, necrosis, and inflammation of vessel walls of all types and sizes, especially small vessels such as postcapillary venules. Among small vessel vasculitides, cutaneous leukocytoclastic vasculitis (LV) is the most common [1]. LV may be idiopathic, or caused by viral, bacterial, and parasitic infections or by vaccines, insect bites, drugs, chemicals, toxins, rheumatologic diseases, or systemic diseases such as cancer [2, 3]. Infections, drugs, and malignant diseases are the most common causes of LV [4], but even with additional testing, identification of the particular etiologic agent can be difficult. LV may, rarely, result from inflammatory bowel disease and cryoglobulinemia. The association between ischemic colitis and LV has not been reported in the literature. The rare cases of LV associated with gastroenterologic diseases have no established standard for the treatment. This paper presents three cases of cutaneous LV, each associated with a different gastroenterologic condition: ischemic colitis, Crohn's disease, and chronic hepatitis C. The treatment of LV caused remission in all of them, regardless of the underlying disease.

**Case 1**

**Figure 1:**
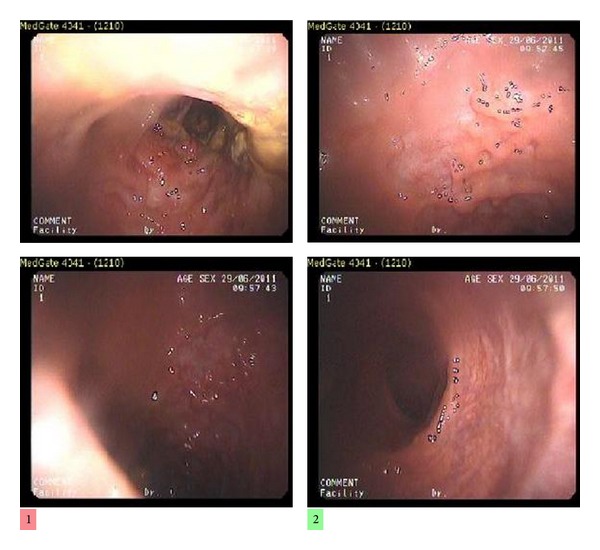
Colonoscopic image.

A 73-year-old male patient suffering from bloody diarrhea that had begun 3 weeks before was referred to us. He also had purpura-like rash on both lower extremities. In his history, he had been diagnosed with hypertension and pulmonary thromboembolism one year before. Our physical examination revealed widespread abdominal tenderness without defender or rebound. Stool microscopy showed an abundance of leukocytes and erythrocytes; his body temperature was 37.3°C. Laboratory test results were normal except for his C-reactive protein (CRP) and erythrocyte sedimentation rate (ESR) (Table 1). The patient's stool culture proved negative, so a colonoscopy was performed. It revealed severe colitis beginning from the distal sigmoid colon and reaching to the midtransverse colon, suggestive of ischemic colitis (Figure 1). The biopsy taken from the column showed widespread ulceration, hemorrhage, and necrosis of the ulcer floor, plus intense fibrinopurulent inflammation in the tissue and the lumen. A computed tomography (CT) angiography showed splenic and portal vein thrombosis, wall thickening of the colon segments from the level of splenic flexure to the rectum, and increasing density in pericolon adipose tissue. The patient was diagnosed with ischemic colitis, and low-molecular-weight heparin treatment was started: enoxaparin sodium (120 mg/day).

Vasculitis was considered in the patient due to increasing CRP (to 17 mg/dL), neutropenia, continuous fever, and purpura in the bilateral lower extremities. The skin biopsy results were compatible with leukocytoclastic vasculitis. As a secondary cause of the vasculitis, coexistence of ischemic colitis and LV was investigated. Treatment began with methyl prednisolone (40 mg/day). On the third day of the steroid therapy, the patient's general condition improved, rashes disappeared, and the patient became afebrile, and, on the fifth day of the steroid therapy, his diarrhea was resolved. The patient was discharged on reduced steroid doses to be followed as an outpatient.

**Case 2**

A 28-year-old male patient had complaints of pain in his stomach and wrists for 2 weeks. His history includes no complaints except for periodic abdominal pain. Physical examination revealed widespread abdominal tenderness, plus tenderness, swelling, and limited range of motion by palpating of elbows, as well as petechial rash on bilateral lower extremities from ankle to kneecap. Selected laboratory tests results are as follows. CRP was 12.34 mg/dL (<0.5 mg/dL), white blood cell (WBC) was 25 × 10^3^/*μ*L, and stool microscopy showed an abundance of leukocytes and erythrocytes. Other laboratory tests were normal (Table 1). An abdominal CT scan showed marked wall thickening along long segments in the jejunal loops, multiple lymphadenopathy (the largest was 2 cm), and bilateral chronic sacroiliitis. Since colonoscopy and biopsy results were compatible with Crohn's disease, treatment was started with methyl prednisolone (60 mg/day), ciprofloxacin (1000 mg/day), and metronidazole (1500 mg/day). During followup, both WBC (15 × 10^3^/*μ*L) and CRP (7 mg/dL) decreased, but skin rashes on the lower extremity did not disappear, and the skin biopsy of the patient showed LV. Secondary reasons for LV such as drug use, infection, and additional diseases were investigated, but none of them was found, so LV was considered to be secondary to inflammatory bowel disease (Crohn's disease).

When the existing methyl prednisolone treatment did not reduce leukocytoclastic vasculitis-related complaints, pulse steroids (methyl prednisolone 1 g/day) were added. After three days, the rashes had regressed so that the patient's treatment was continued with oral steroids.

**Case 3**

**Figure 2:**
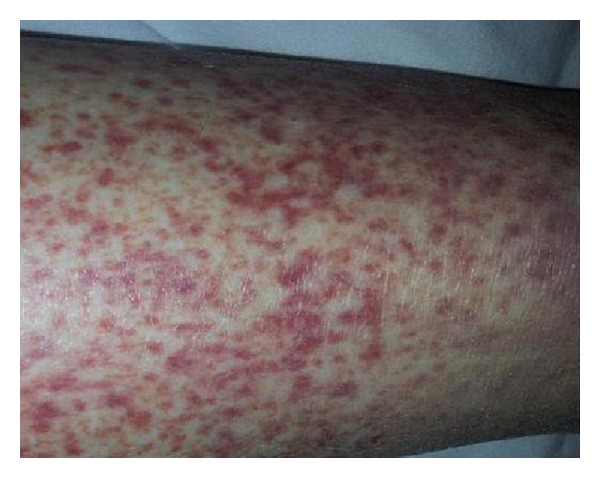
Cutaneous leukocytoclastic rash.

A 59-year old female patient was admitted with complaints of rashes on her legs for 2 months and bleeding of the nose that had started 2 days before. In her history, the patient had been diagnosed for chronic hepatitis C (HCV), but went untreated for 2.5 years as there had been no indication to require any treatment. Physical examination was unremarkable except for petechial rashes on her bilateral lower extremities. Laboratory tests detected the following: platelet, 18 × 10^3^/*μ*L, anti-HCV (+), cryoglobulin (+), and complement C4 <1.47 mg/dL. According to clinical and laboratory results, cryoglobulinemia due to chronic hepatitis C was diagnosed (Table 1). Because of the rashes on her legs, vasculitis was considered, which a punch biopsy helped to definitively diagnose (Figure 2). Without treating the HCV, steroid therapy was initiated for LV. Clinical examination and laboratory findings for LV improved. After three days of methyl prednisolone therapy (60 mg/day), platelet values had increased and rashes had decreased. With this improvement, the patient was discharged to be followed up in the gastroenterology clinic, and after one month, methyl prednisolone therapy was interrupted. When the rash returned on her legs and the platelet values had again decreased, the patient was again admitted to the hospital and put on methyl prednisolone (32 mg/day) with the addition of azathioprine (50 mg/day). When the patient complaints declined, she was discharged to be followed up in the outpatient clinic.

## 2. Discussion

Leukocytoclastic vasculitis is a pathological condition first defined in 1950 by Pearl Zeek as vasculitis of small vessels after drug intake. LV is characterized by exudates rich in neutrophils, endothelial damage, fibrin deposition, and core fragments (leukocytoclasis) in postcapillary venules of small vessels. Patients diagnosed with LV who have isolated skin involvement but no internal organ involvement are considered to have cutaneous LV [5, 6]. Approximately 23% of the cases of cutaneous LV are associated with infections, 20% with drugs, 12% with connective tissue diseases, and 4% with malignancies. Cutaneous LV is not common in primary systemic vasculitis such as Wegener's granulomatosis, polyarteritis nodosa, microscopic polyangiitis, and Churg-Strauss syndrome and generates only 4% of all cases of cutaneous LV. In the literature, the cause of cutaneous LV has been reported as idiopathic in 3% and 72% of cases [7, 8]. Rarely, inflammatory bowel disease, cryoglobulinemia, and bowel bypass syndrome may be the cause of cutaneous LV.

The association between ischemic colitis and LV has not yet been reported in the literature. We wanted to present Case 1 because ischemic colitis was a definitive diagnosis and other causes of LV were excluded. However, we could find no underlying cause of the ischemic colitis. Whether LV developed due to ischemic colitis or ischemic colitis developed because of underlying LV is unclear, but in either case anticoagulant therapy produced no response. Clinical and laboratory improvement of the ischemic colitis was verified by the treatment of LV. Perhaps steroid therapy had more response because ischemic colitis developed secondary to LV.

Treatment of cutaneous LV is based on the degree of systemic involvement and should be appropriate to the underlying disease. Most patients have only scattered purpuric lesions and, clinically, no systemic involvement. Rashes usually restrict themselves. In the treatment of LV, if the encountering drug and antigen are eliminated, symptoms will disappear without treatment within days or weeks. Symptomatic treatment is given; bed rest is recommended. Any underlying infection should be treated. Patients who have long-standing skin manifestations, severe cutaneous involvement, and/or systemic disease should be treated with oral or parenteral corticosteroids. Prednisolone (20–60 mg daily in divided doses) will control the disease. Dosage should be gradually reduced to the lowest possible amount, and then the treatment must be terminated [9, 10]. In Case 2, however, steroid therapy, the major treatment for both Crohn's disease and LV, was not effective, so pulse steroid therapy was started, and then the rashes vanished.

Low levels of serum complement, positive serum cryoglobulins, and high ESR may call for a diagnosis of cryoglobulinemia secondary to chronic hepatitis C [11], such as in Case 3, who also had LV + cryoglobulinemia + HCV. The LV was treated with steroid alone, without at the same time addressing the HCV. Steroid therapy resulted in remission of LV, but when steroid dose was reduced, LV recurred. Because of the risk of activating the hepatitis C infection, we kept to the lowest possible steroid dosage, therefore, azathioprine therapy was added to the initial course of treatment. By such means, remission was achieved again. The patient is still in remission, and her hepatitis C has not activated.

In summary, LV cases due to ischemic colitis, Crohn's disease, and chronic hepatitis C are rarely seen in practice and have no standard treatment. Each case may require separate treatment protocols, as did our three. Regardless of the underlying disease, however, these patients' clinical and laboratory abnormalities resolved completely by being treated only for leukocytoclastic vasculitis.

## Figures and Tables

**Table 1:** Laboratory examination of three cases.

	Case 1	Case 2	Case 3
White blood cell (10^3^/*µ*L)	6.57	25.56	2.63
Neutrocytes (%)	80.9	88	64.6
Lymphocytes (%)	12.2	10	21.7
Hemoglobin (g/dL)	10.7	15.2	10.6
Platelets (10^3^/*µ*L)	212	130	18
C-reactive protein (mg/dL) (0.5<)	9.76	12.34	0.14
C4 (mg/dL) (10–40)	n.t.	n.t.	<1.47
Anti-HCV (positive > 1.0 COI)	(−)	(−)	208.1
Cryoglobulin	(−)	(−)	(+)
Skin biopsy	LV	LV	LV

Case 1: ischemic colitis, Case 2: Crohn's disease, and Case 3: cryoglobulinemia due to chronic hepatitis C, leukocytoclastic vasculitis (LV), not tested (n.t.), and anti-hepatitis C virus (Anti-HCV).

## References

[1] Hautmann G, Campanile G, Lotti TM (1999). The many faces of cutaneous vasculitis. *Clinics in Dermatology*.

[2] Cassidy JT, Petty RE, Casidy JT, Petty RE, Laxer RM, Lindsly CB (2005). Leukocytoclastic vasculitis. *Textbook of Pediatric Rheumatology*.

[3] Rostoker G (2001). Schönlein-Henoch purpura in children and adults: diagnosis, pathophysiology and management. *BioDrugs*.

[4] Wilmoth GJ, Perniciaro C (1996). Cutaneous extravascular necrotizing granuloma (Winkelmann granuloma): confirmation of the association with systemic disease. *Journal of the American Academy of Dermatology*.

[5] Tsai TF, Chen RL, Su IJ (1994). Epstein-Barr virus-associated lymphoproliferative disorder of granular lymphocytes presenting initially as cutaneous vasculitis. *Journal of the American Academy of Dermatology*.

[6] Jennette JC, Falk RJ, Andrassy K (1994). Nomenclature of systemic vasculitides: proposal of an international consensus conference. *Arthritis & Rheumatism*.

[7] Gross RL, Brucker J, Bahce-Altuntas A (2011). A novel cutaneous vasculitis syndrome induced by levamisole-contaminated cocaine. *Clinical Rheumatology*.

[8] Garcia-Porrua C, Gonzalez-Gay MA (1999). Comparative clinical and epidemiological study of hypersensitivity vasculitis versus Henoch-Schonlein purpura in adults. *Seminars in Arthritis and Rheumatism*.

[9] Carlson JA, Ng BT, Chen K-R (2005). Cutaneous vasculitis update: diagnostic criteria, classification, epidemiology, etiology, pathogenesis, evaluation and prognosis. *American Journal of Dermatopathology*.

[10] Monti G, Galli M, Invernizzi F (1995). Cryoglobulinaemias: a multi-centre study of the early clinical and laboratory manifestations of primary and secondary disease. GISC. Italian Group for the Study of Cryoglobulinaemias. *Quarterly Journal of Medicine*.

[11] Chung L, Funke AA, Chakravarty EF, Callen JP, Fiorentino DF (2006). Successful use of rituximab for cutaneous vasculitis. *Archives of Dermatology*.

